# Apoptosis is increased and cell proliferation is decreased in out-of-phase endometria from infertile and recurrent abortion patients

**DOI:** 10.1186/1477-7827-8-126

**Published:** 2010-10-22

**Authors:** Gabriela F Meresman, Carla Olivares, Susana Vighi, Margarita Alfie, Marcela Irigoyen, Juan J Etchepareborda

**Affiliations:** 1 Institute of Biology and Experimental Medicine (IBYME - CONICET), Vuelta de Obligado 2490, (C1428ADN) Ciudad Autónoma de Buenos Aires, Argentina; 2 Anatomy-pathology Department, José de San Martín Hospital, Av. Córdoba 2351 (C1120AAR) Ciudad Autónoma de Buenos Aires, Argentina; 3 Sterility Sector, Division of Gynaecology, José de San Martín Hospital, Av. Córdoba 2351 (C1120AAR) Ciudad Autónoma de Buenos Aires, Argentina

## Abstract

**Background:**

Various endometrial abnormalities have been associated with luteal phase deficiency: a significant dyssynchrony in the maturation of the glandular epithelium and the stroma and a prevalence of out-of-phase endometrial biopsy specimens. Out-of phase endometrium is a controversial disorder related to failed implantation, infertility and early pregnancy loss. Given that the regulation of the apoptotic process in endometrium of luteal phase deficiency is still unknown, the aim of this study was to evaluate cell proliferation, apoptosis and the levels of the main effector caspase, caspase-3 in the luteal in-phase and out-of-phase endometrium.

**Methods:**

Thirty-seven endometrial samples from sterile or recurrent abortion patients were included in this study: 21 in-phase samples (controls) and 16 samples with out-of-phase endometrium. Biopsy specimens of eutopic endometrium were obtained from all subjects during days 21-25 of the menstrual cycle. The endometrium with endometrial maturity of cycle day 25 or less at the time of menstruation was considered out-of phase. Endometrial tissues were fixed in 10% buffered formaldehyde. For apoptosis quantification, sections were processed for in situ immunohistochemical localization of nuclei exhibiting DNA fragmentation, by the terminal deoxynucleotidyl transferase (TdT)-mediated dUTP digoxygenin nick-end labeling (TUNEL) technique. Expressions of Proliferating Cell Nuclear Antigen (PCNA) as a marker of cell proliferation, and of cleaved caspase-3 as a marker of apoptosis, were assessed by immunohistochemistry in the luteal in-phase and out-of-phase endometrium from infertile and recurrent abortion patients.

**Results:**

Luteal out-of-phase endometrium had increased apoptosis levels compared to in-phase endometrium (p < 0.05). Caspase-3 evaluation confirmed these results: the luteal out-of-phase endometrium showed augmented cleaved caspase-3 expression (p < 0.005). As well, our data demonstrated that the luteal out-of-phase endometrium expresses decreased PCNA levels (p < 0.05), showing that cell proliferation is diminished in this tissue.

**Conclusions:**

this study represents the first report describing variations at the cell proliferation and cell death levels in the out-of-phase endometrium in comparison with in-phase endometrium from infertile and recurrent abortion patients. Further studies are needed to elucidate a potential role of these alterations in the physiopathology of luteal phase deficiency.

## Background

Endometrial remodelling occurs during each menstrual cycle in women. The secretory activity in the second half of the menstrual cycle is characterized by a diversity of structural changes, showing a different pattern throughout the cycle [1]. During the luteal phase, the endometrium is under direct stimulation by progesterone (P). A rapid decline in P or an inadequate P concentration during this period results in a degenerative endometrium, which is not receptive for implantation of a fertilized ovum or maintenance of early pregnancy [2].

Luteal phase defect (LPD) is a controversial syndrome believed to be related to failed implantation, infertility and early pregnancy loss [3-6]. The physiopathology of LPD involves disorders such as a luteal phase of less than 10 days, abnormal luteinization that causes a decrease in androstenedione and abnormal follicular development [7,8]. In stimulated in vitro fertilization (IVF) cycles, the main cause of the LPD has been related to the multifollicular development achieved during ovarian stimulation [9].

Various endometrial abnormalities have been associated with LPD: a significant dyssynchrony in the maturation of the glandular epithelium and the stroma and a prevalence of out-of-phase endometrial biopsy specimens [1]. However, it is difficult to establish the exact incidence of out-of phase endometrium and of LPD because the assessment of histological dating is frequently subjective and lacks precision [10-12].

For many years, endometrial dating was an accepted assay of the quality of luteal function and a diagnostic test for LPD. However, recently, the accuracy and reproducibility of endometrial dating have been challenged [4]. Indeed, the diagnosis of LPD in the clinical setting remains problematic and controversial primarily because there is no practical diagnostic method that has been unquestionably validated.

Out-of-phase endometrium is an aberration that is often found in infertile patients. Even if it is well known that abnormal endometrium is an important cause to recurrent miscarriage, Peters et al. found no significant differences between fertile, infertile and recurrent pregnancy loss patients with in- and out-of-phase endometrium [13]. At present, the origin of out-of-phase remains controversial.

Apoptosis, or programmed cell death, plays an important role in the cyclic changes that take place during the menstrual cycle. This mechanism is coded genetically and contributes to the homeostasis of the tissues. Several investigations have revealed that uterine endometrium can be regulated by apoptosis [14-16]. In the human endometrium, there had been shown changes in apoptosis of the glandular epithelium throughout the menstrual cycle [17,18]. Recent publications confirmed the presence of endometrial apoptosis, mainly in the late secretory phase of the menstrual cycle [14,15].

Apoptosis is an important mechanism in the regulation of the endometrial growth both in the physiology and in the pathology. Previous work from our group, showed an increased survival capability in the eutopic endometrium from patients with endometriosis. This abnormal survival of endometrial cells may result in their continuing growth into ectopic locations [19-21]. In addition, it is well known that the regulation of placental apoptosis is essential for the normal physiology of pregnancy during implantation since apoptosis is important for the appropriate tissue remodelling of the maternal decidua and invasion of the developing embryo [22].

Given that the regulation of the apoptotic process in endometrium of LPD is still unknown, the aim of this study was to evaluate cell proliferation, apoptosis and the levels of the main effector caspase, caspase-3 in the luteal in-phase and out-of-phase endometrium.

## Methods

### Tissue collection

Fifty five endometrial samples were obtained from patients who attended for sterility or recurrent abortion at the Sterility Sector of Clinics Hospital from Buenos Aires. Eighteen samples were excluded as 3 were dissociated endometrium, 10 were proliferative, 3 became pregnant at that cycle and 2 had endometritis. Overall, a total of 37 luteal endometrial samples were included in this study. All patients involved gave informed consent to participate in the present study, which has approved by the Hospital and the Institutional Ethics Committees.

The control group consisted of 21 in-phase luteal endometrium samples and the study group consisted of 16 luteal out-of-phase endometrium samples. Most of the patients attended for sterility, only 3 patients in the control group and 2 in the out of phase group attended for early recurrent abortion. Both groups were similar in age (33,3 ± 1.0 and 35,3 ± 0.9 years in average respectively). Underlying pathology was discarded in all cases by histological and clinical studies.

Biopsy specimens of eutopic endometrium were obtained from all subjects during days 21-25 of the menstrual cycle using a Novak curette. For endometrial dating, 4 μm sections stained with hematoxylin and eosin and periodic acid Schiff stain were evaluated. All endometrial biopsies were evaluated by the same expert pathologist according to the histopathological criteria of Noyes *et al *[23]. The endometrium with endometrial maturity of cycle day 25 or less at the time of menstruation was considered out-of phase. Endometrial tissues were fixed in 10% buffered formaldehyde.

### Apoptosis detection system

For apoptosis quantification, sections were processed for in situ immunohistochemical localization of nuclei exhibiting DNA fragmentation, by the TUNEL technique, using the apoptosis detection kit Apoptag Plus (Chemicon International, Temecula CA, USA). Sections were treated according to the manufacturer's instructions as previously described [19].

Briefly, sections were deparaffinized and rehydrated with xylene and ethanol, and permeabilized with 20 μg/ml Proteinase K (Gibco, Grand Island, NY, USA). Endogenous peroxidase was inactivated by coating the samples with 3% H_2_O_2_. Sections were rinsed with PBS, and then immersed 60 min in TdT buffer at 37°C. Afterwards, they were incubated 30 min with the anti-digoxygenin peroxidase conjugate, followed by the peroxidase substrate diaminobenzidine (DAB). Finally, sections were counterstained with hematoxylin. Sections of female rodent mammary gland obtained 3-5 days after weaning of pups, were used as a positive control. As a negative control, a number of tissue samples were subjected to treatment without TdT. The percentage of apoptotic cells was determined by counting labeled cells at 400X magnification in 10 randomly selected and homogeneous fields.

Apoptotic cells were also identified by their characteristic morphological features in hematoxylin-eosin-stained endometrial sections: cell shrinkage and chromatin margination or chromatin condensation with formation of apoptotic bodies [19].

The TUNEL method is characterized by higher sensitivity than most other histochemical approaches and is considered to be the gold standard to detect apoptosis in situ [24]. However, in order to complete the apoptosis evaluation we also included the assessment of the cleaved caspase-3, the most important effector enzyme in apoptosis pathway.

### Immunohistochemistry

Serial sections of the endometrium were subjected to standard immunohistochemistry as previously described [25]. Briefly, sections were deparaffinized in xylene and rehydrated through graded alcohols followed by microwaving in 0.01 M sodium citrate buffer for antigen retrieval. Endogenous peroxidase was blocked by treatment with 3% hydrogen peroxide for 10 min at room temperature, after which, non-specific binding was blocked incubating with 4% bovine serum albumin in phosphate buffer for 60 min. Tissue sections were incubated overnight with the primary antibody: rabbit anti-human PCNA polyclonal (FL-261, Santa Cruz Biotechnology, Santa Cruz, CA); or rabbit anti-human cleaved caspase-3 (CP 229, Biocare Medical, Concord, CA) at 4°C. After that, sections were treated for 60 min with a universal secondary biotinylated antibody followed by incubation with streptavidin-peroxidase conjugated (LSAB+ Kit/HRP System, Dako, Carpinteria, CA). Binding was visualized by incubating sections with DAB and lightly counterstaining with hematoxylin, prior to permanent mounting. As a negative control, species and class matched igG was used. The negative control showed absence of specific staining.

PCNA positive cells were identified by the presence of brown nuclear reactivity in the endometrial epithelial cells. PCNA, also called cyclin, is a 36-Kd auxiliary protein of DNA polymerase-delta, which was found to be a useful marker in immunocytochemical studies of cell proliferation, because its expression correlates with the proliferative state of the cell [26]. PCNA is a proliferation marker for cells in early G1 phase and S phase of the cell cycle. PCNA immunohistochemistry has been extensively used for basic research and as a prognostic tool in surgical pathology [27-29] and has good correlation with KI-67 [30] expression. It has been shown that immunohistochemistry, to detect nuclear antigens such as PCNA expressed during the cell cycle in proliferating cells, is a good approach to assess cell proliferation [31].

The number of cells expressing immunoreactivity for PCNA per 100 cells was established using a standard light microscope by two independent observers. The total number of epithelial cells in 10 representative fields was counted. Any nuclear staining was regarded as positive. There was no significant difference in results between the two observers (data not shown).

Cleaved caspase-3 detects endogenous levels of the large fragment (17/19 kDa) of activated caspase-3 resulting from cleavage adjacent to (Asp175). Activation of caspase-3 requires proteolytic processing of its inactive zymogen into active p17 and p12 subunits. Staining for cleaved caspase-3 was assessed to study apoptosis in endometrial epithelial cells. The percentage of cells expressing immunoreactivity for cleaved caspase-3 was established by analyzing 10 representative fields from each specimen.

### Statistics

Statistical analysis of the data was performed using GraphPad Instat V4.0 software. A non-parametrically two-tailed Mann-Whitney U-test was used for determination of differences in the two groups. Results were expressed as mean ± S.E.M. A value of p < 0.05 was considered to be significant.

## Results

### Apoptosis in in-phase and out-of-phase endometrium

**Figure 1:**
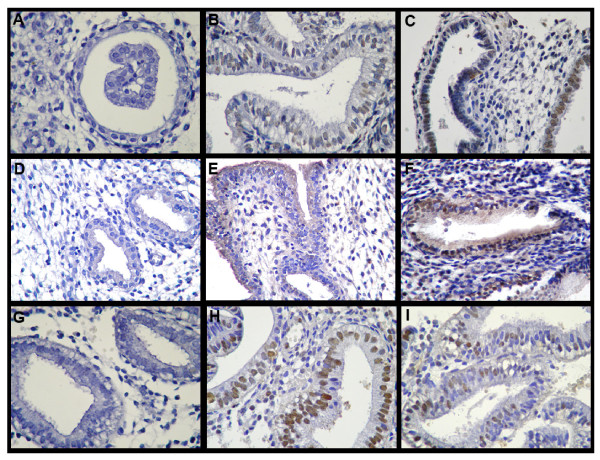
**Apoptosis, cleaved caspase-3 expression and cell proliferation in in-phase and out-of-phase endometrium**. Histological sections were assessed for apoptosis by TUNEL (A-C) and immunostained for cleaved caspase-3 (D-F) and PCNA (G-I). Negative control sections were incubated in absence of TdT for apoptosis detection (A) and with an immunoglobulin of the same immunoglobulin class and concentration as the primary antibody for cleaved caspase-3 (D) and PCNA expression (G). Out-of-phase endometrium shows higher levels of apoptotic cells (C) and cleaved caspase-3 positive cells (F) and decreased levels of cell proliferation (I) compared to in-phase endometrium (B, E, and H respectively). Magnification: 400X.

**Figure 2:**
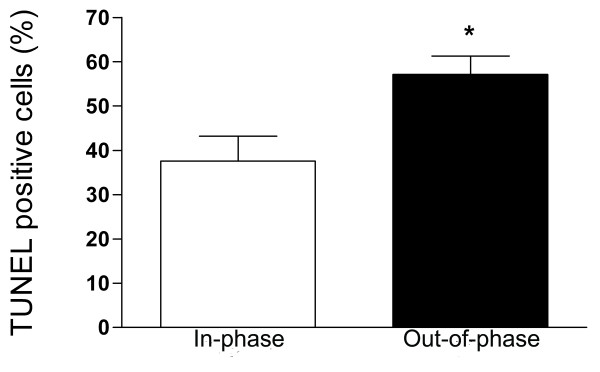
**Apoptosis in in-phase and out-of-phase endometrium**. Apoptosis was assessed by TUNEL technique in the epithelial fraction. The percentage of apoptotic cells were determined by counting labelled cells at 400X magnification in 10 randomly selected and homogeneous fields. Out-of-phase endometrium presents a significantly higher percentage of apoptotic cells than in-phase controls. * p < 0.05 vs. Control.

The apoptosis detection system revealed positive staining only in the glandular epithelium of the endometrium sections. An increased apoptosis was detected in eutopic out-of-phase endometrium compared to in-phase controls: 57.1 ± 4.1 vs. 37.5 ± 5.6 expressed as percentage of TUNEL positive cells (p < 0.05, Figure 1B and 1C and Figure 2).

### Immunohistochemistry for cleaved caspase-3 in in-phase and out-of-phase endometrium

**Figure 3:**
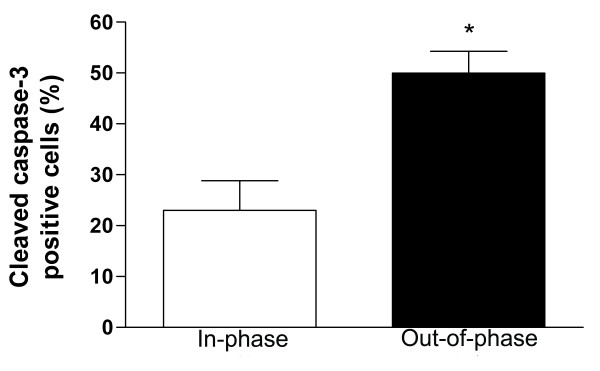
**Cleaved caspase-3 expression in in-phase and out-of-phase endometrium**. Histological sections were immunostained for cleaved caspase-3 expression (see text). Cleaved caspase-3 detects endogenous levels of the large fragment (17/19 kDa) of activated caspase-3. The percentage of cells expressing immunoreactivity for cleaved caspase-3 was established by analyzing 10 representative fields from each specimen. Out-of-phase endometrium shows a significantly higher percentage of cleaved caspase-3 positive cells than in-phase controls. ** p < 0.005 vs. Control.

Complementary to the results obtained by the TUNEL method, the immunohistochemical staining using a specific antibody for the cleaved fragment of caspase-3 revealed increased immunoreactivity in epithelial endometrial cells from out-of-phase samples compared to in-phase controls: 49.9 ± 4.2 vs. 23.0 ± 5.82 expressed as percentage of cleaved caspase-3 positive cells (p < 0.005, Figure 1E and 1F and Figure 3).

### Cell proliferation in in-phase and out-of-phase endometrium

**Figure 4:**
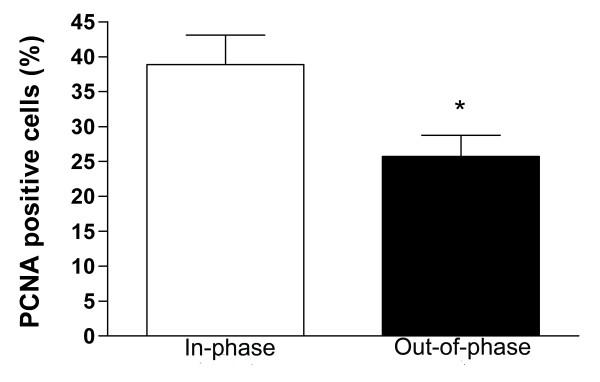
**Cell proliferation in in-phase and out-of-phase endometrium**. Histological sections were immunostained for PCNA expression (see text). The total number of epithelial cells in 10 representative fields was counted. Cell proliferation was quantified in the epithelial fraction as percentage of PCNA positive cells. Out-of-phase endometrium shows a significantly lower percentage of PCNA positive cells than in-phase endometrium. * p < 0.05 vs. Control.

The out-of-phase endometrium showed a significantly decreased degree of cell proliferation in the epithelial cells in comparison to in-phase controls: 25.7 ± 3.0 vs. 38.9 ± 4.2 expressed as percentage of PCNA positive cells (p < 0.05, Figure 1H and 1I and Figure 4).

## Discussion

Luteal phase defect (LPD) is a controversial entity of considerable clinical importance that is implicated in infertility and recurrent spontaneous abortion. A prevalence of out-of-phase endometrial biopsy specimens have been associated with luteal phase deficiency (LPD) [1]. Luteal phase inadequacy is a subtle disruption of luteal function and it may be considered the most common ovulatory disorder in women [32]. It has also been found that most of women with out-of-phase midluteal endometrium may have a cryptic form of LPD [33].

Despite the existence of LPD has been questioned by some authors [34] and a recent report showed that out-of-phase biopsy poorly discriminated between women from fertile and infertile couples in either mid luteal or late luteal phase [35], there has been abundant clinical and experimental research establishing the reality and importance of LPD [4,8,36-38]. Although some authors prefer to perform evaluation of serum P [39], the diagnosis of LPD is best based on a histological study of the endometrium [11]. The most commonly used method to assess luteal function in infertility has been the direct evaluation of endometrial biopsy and histological dating [40]. It was found that an endometrial biopsy which was well dated showed a definite correlation with the P assays and could be considered as the most easily performed and reliable indicator, useful in detecting a luteal phase defect [41].

In order to identify some endometrial differentiation patterns between in- and out-of phase-endometrium, Hirama and Ochiai examined the role of hormone receptor compartmentalization in infertile women and found that the level of cytosol estrogen receptor was significantly lower in out-of-phase endometrium regardless of serum P levels [42]. In addition, differences in integrin expression between in- and out-of-phase endometrium were observed for αvβ3 integrin [40].

For the purpose of continuing with the identification of the distinctiveness between in-phase and out-of phase endometrium, the aim of the present study was to investigate whether apoptotic endometrial characteristics differ in samples with or without this abnormality. Endometrial cell death will be menstrual cycle dependent, and therefore timing of biopsy is crucial. The endometrial biopsies used in this study were from days 21-25 of the menstrual cycle.

In the present study we found that the out-of-phase endometrium from infertile and abortion patients had increased apoptosis levels. In addition, caspase-3 evaluation confirmed these results: the out-of-phase endometrium derived from these patients showed augmented cleaved caspase-3 expression, which indicated caspase-3 activation at the epithelial cells compared to in-phase endometrium. Furthermore, our data demonstrated that the out-of-phase endometrium from infertile and abortion patients expresses decreased PCNA levels, showing that cell proliferation is diminished in this endometrial tissue.

In this work, we decided to evaluate glandular apoptosis of in-phase and out-phase endometrium since it was reported in several works that endometrial epithelial glandular cells show the most significant cyclical apoptotic changes throughout the cycle. Apoptotic cells were mainly detected in the glandular epithelium of the endometrium and only very few apoptotic cells were detected in the stroma at any stage of the cycle. The endometrial cells in the basal layer showed no evidence of apoptosis throughout the menstrual cycle [43]. In addition, Bcl-2, Fas and FasL were found predominantly in the endometrial glandular fraction and changed dramatically during the menstrual cycle, while in stromal cells these apoptosis related proteins showed no expression or not significant cyclic changes [16,44,45]. Also, expression of Bax and Bcl-x was predominantly localized to epithelial cells of the functionalis layer of the secretory endometrium and Tao et al. concluded that cyclic changes in endometrial growth and regression may be precisely regulated by shifts in the ratio or balance of Bcl-2 and related proteins in glandular epithelial cells [18]. In addition, it has been reported that Ki-67, a proliferation marker, is expressed specifically in glandular epithelial cells [46]. Taken as a whole, we came to the decision that the proper way of studying the apoptosis status of the endometrium was evaluating the apoptosis rate of the epithelial cells.

Although in the present work the P levels were not included since the selection criteria of the out-of-phase endometrium was based on the histological dating, a direct correlation between the decrease of P levels and the increase of apoptosis has been demonstrated by other authors. However, a controversy exists at the time since some scientists claimed that histological endometrial dating does not reflect circulating P concentrations, and that decreased progesterone receptors on endometrial gland nucleus results in a deficient response of endometrium to proper stimulus of progesterone. It has been reported that LPD is more likely to be a result of an abnormal response of the endometrium to P, than to a subnormal production of P by the corpus luteum [4,11].

It is well known that endometrial cell proliferation and cell death are regulated by ovarian hormones. In the endometrium, the fall of ovarian P in late secretory phase or the withdrawal of ovarian hormones, are followed by apoptosis. Removal of P led to a substantial increase of endometrial apoptosis, to a significant induction of the proapoptotic proteins Fas, FasL, BIM expression and to an increase of the bcl-X_S_/bcl-X_L _ratio [47-49].

Additionally, apoptosis is an important component of the correct implantation process [22]. Although in the present paper increased endometrial cell death rates were observed in out-of-phase endometrium samples, a direct correlation with spontaneous pregnancy loss or infertility has not been demonstrated. Nevertheless abundant data illustrating this association have been reported in the literature. In pregnancies complicated by preeclampsia or intrauterine growth restriction, there is an augmented incidence of placental apoptosis which is associated with deficient trophoblast invasion [50,51]. In addition, elevated apoptosis rates were found in abortion-prone mice [52] and a strong increase of Bax expression was found in the cytotrophoblast, stroma, endothelial cells and deciduas of placentas of the first trimester abortion compared to the low/moderate Bax immunopositivity in all the placental compartments during the first trimester after voluntary termination of pregnancy [53]. FasL and Fas are also elevated in the endometrium and in endometrial lymphocytes associated with abortion in experimentally model of porcine spontaneous fetal loss [54]. As well, Savion et. al. suggested a possible role for the apoptotic process in mechanisms mediating pregnancy loss and indicated an involvement of p53 and bcl-2 in its regulation [55]. All these data suggest that infertility or recurrent abortion in LPD patients with out-of-phase endometrium could be associated to the increased cell death levels observed in their endometrial samples.

One of the limitations of our study is that there is no comparison with endometrium from normal fertile women. However, underlying pathology was discarded in all cases by histological and clinical studies. Furthermore, the purpose of this study was to address the question about differences between in-phase and out-of-phase endometrium and inclusion of endometrium samples from normal fertile patients was not essential for this intention.

Additional evidence is necessary to know if infertility or recurrent abortion in LPD patients with out-of-phase endometrium could be associated to the increased cell death levels observed in their endometrial samples.

## Conclusions

In conclusion, this study represents the first report describing variations at the cell proliferation and cell death levels in the out-of-phase endometrium from infertile and abortion patients in comparison with in-phase endometrium. Further studies are needed to elucidate a potential role of these alterations in the physiopathology of LPD.

## Competing interests

The authors declare that they have no competing interests.

## Authors' contributions

GM designed the study, performed the experiments, participated in discussion of the results and drafted the manuscript. CO performed the experiments and participated in discussion of the results. SV is an expert gynaecologic pathologist who confirmed the diagnosis of out-of phase endometrium. MA and MI provided the patient samples and were responsible for supervision of this work. JJE was responsible for the conception, design, discussion of the results, drafting and critical revision of the manuscript. All authors read and approved the final manuscript.
